# Case of pediatric cerebellar, hippocampal, and basal nuclei transient edema with restricted diffusion (CHANTER) syndrome in a 2-year-old girl

**DOI:** 10.1007/s00247-024-05928-2

**Published:** 2024-04-17

**Authors:** Robert A. Koenigsberg, Luke Ross, Jason Timmerman, Rithika Surineni, Kara Breznak, Tina C. Loven

**Affiliations:** 1 Department of Radiology, Temple University Hospital, Philadelphia, PA USA; 2 Saint Christopher’s Hospital for Children, Philadelphia, PA USA

**Keywords:** Cerebellar infarction, CHANTER, Opioid, Child

## Abstract

Cerebellar, hippocampal, and basal nuclei transient edema with restricted diffusion (CHANTER) syndrome is a recently described entity that refers to a specific pattern of cerebellar edema with restricted diffusion and crowding of the fourth ventricle among other findings. The syndrome is commonly associated with toxic opioid exposure. While most commonly seen in adults, we present a case of a 2-year-old girl who survived characteristic history and imaging findings of CHANTER syndrome.

## Introduction

Cerebellar, hippocampal, and basal nuclei transient edema with restricted diffusion (CHANTER) syndrome represents a rare disorder with a constellation of imaging findings in patients with opioid neurotoxicity. Clinically, patients present with altered mental status in the context of substance intoxication. The recently described pattern of imaging findings includes cytotoxic edema in bilateral hippocampi, cerebellar cortices, and the basal ganglia [1]. Patients also frequently develop early obstructive hydrocephalus from fourth ventricular compression [2]. Although these findings are usually associated with irreversible neuronal death and poor overall outcomes, patients with CHANTER syndrome can show significant clinical and radiographic improvement over time [1]. The disease has almost exclusively been observed and reported in adults, but we present the case of a 2-year-old girl who was found unconscious after consuming fentanyl. Her clinical presentation, history, and subsequent imaging findings are all consistent with a diagnosis of CHANTER syndrome.

## Case description

**Fig. 1:**
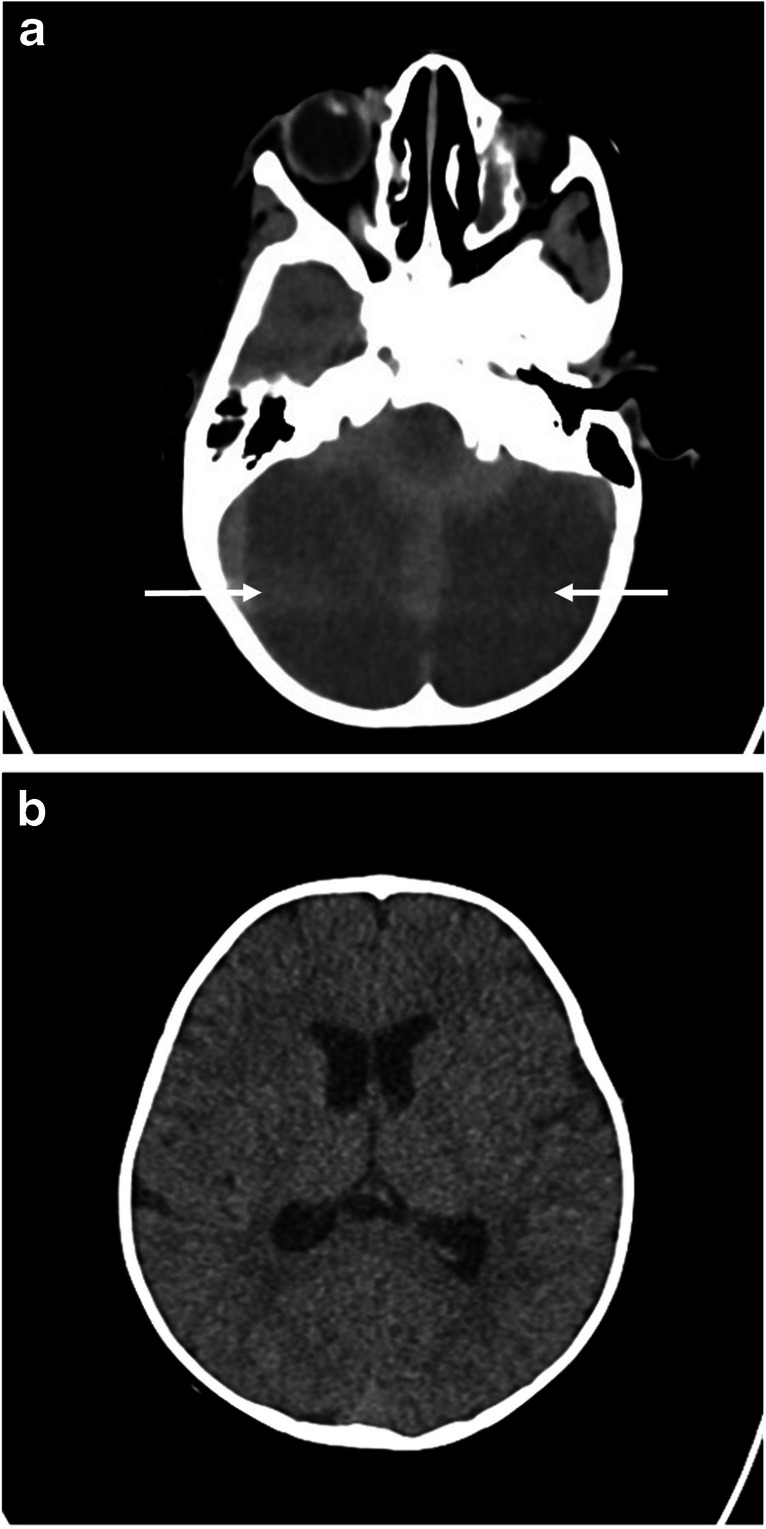
Two-year-old girl found unresponsive after fentanyl ingestion. **a** Axial noncontrast CT head at the level of the posterior fossa shows bilateral diffuse cerebellar hypodensity suggestive of diffuse edema or infarction. **b** Mild ventriculomegaly is noted at the level of the lateral ventricles

A 2-year-old female was found unconscious in her home for an unknown length of time. Urine drug screen was positive for fentanyl and a respiratory panel was positive for influenza. Noncontrast head computed tomography (CT) performed at day of presentation showed diffuse hypodensity in the bilateral cerebellar hemispheres and early ventriculomegaly (Fig. 1a, ab).

**Fig. 2:**
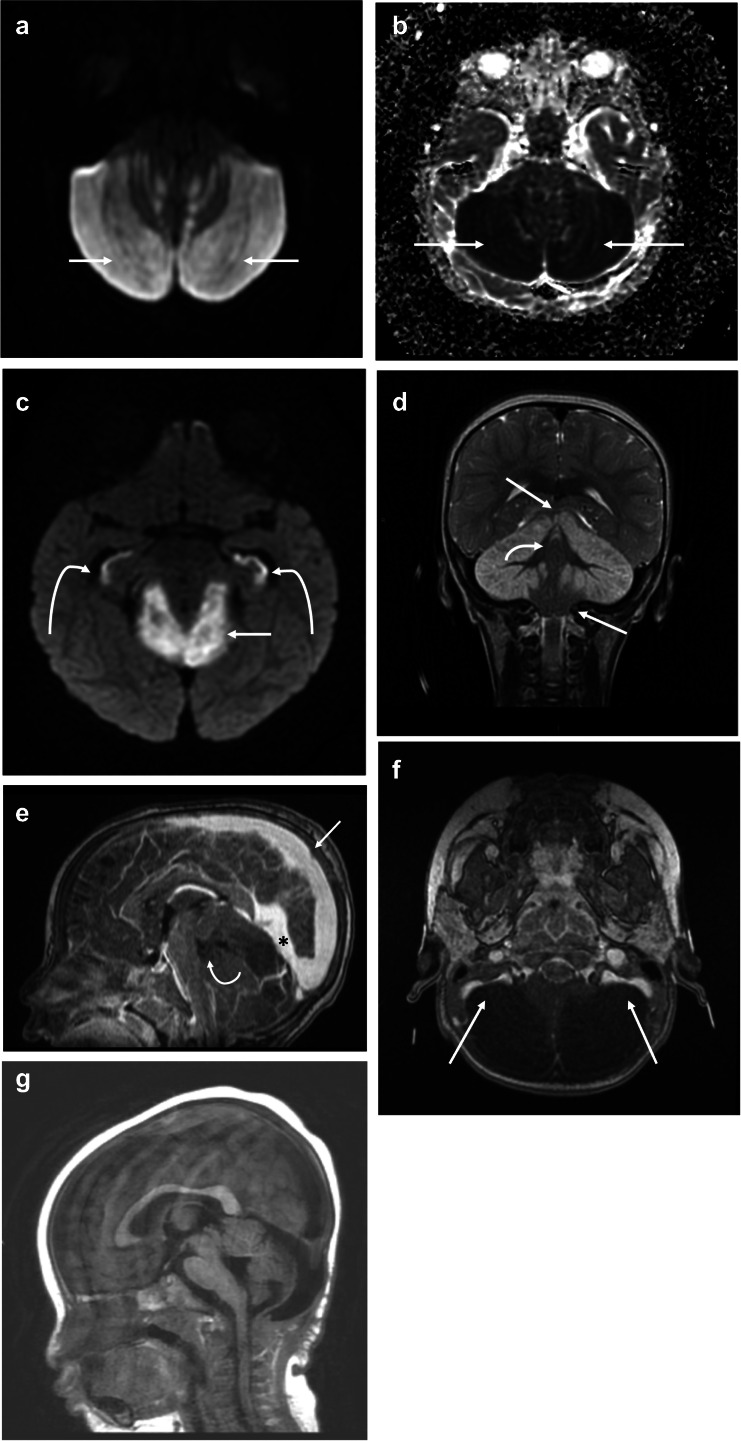
**a** Axial diffusion-weighted image (DWI) b1,000 s/mm^2^ showing diffuse cerebellar restricted diffusion confirmed as profound decreased signal on ADC map (**b**). **c** Axial DWI shows symmetric hippocampal restricted diffusion (curved arrows). Superior transtentorial herniation with cerebellar cortical restricted diffusion (straight arrow). **d** T2-weighted coronal image shows diffuse cortical cerebellar hemispheric edema with superior transtentorial herniation and bilateral downward cerebellar tonsillar herniation (straight arrows). Notice relative sparing of edema in cerebellar vermis (curved arrow). **e** Sagittal contrast T1 midline image demonstrating severe brainstem compression. Notice severely dilated sagittal (arrow) and straight (*) dural sinuses and compression of fourth ventricle (curved arrow). **f** Axial contrast T1 image at the level of the medulla confirming symmetric bilateral sigmoid sinus compression (arrows). **g** Follow-up T2 axial image obtained 2 months after initial presentation shows bilateral cerebellar encephalomalacia

Subsequent brain magnetic resonance imaging (MRI) showed worsening of cerebellar edema, mass effect, and cerebellar herniation. There was bilateral restricted diffusion involving the cerebellum confirmed on apparent diffusion coefficient ADC map (Fig. 2a and b). Bilateral restricted diffusion was also demonstrated separately within the hippocampus bilaterally (Fig. 2c). Coronal T2 imaging demonstrated diffuse cerebellar edema with superior transtentorial herniation and downward tonsillar herniation at the level of the foramen magnum (Fig. 2d). Magnetic resonance angiography (MRA) of the brain was performed which showed normal caliber of the intracranial arteries, including the vertebral arteries at the skull base (not shown). However, there was proximal dural venous sinus distention due to bilateral sigmoid sinus compression from bilateral cerebellar edema (Fig. 2e and f).

Following MRI, the patient was emergently taken to the operating room for suboccipital decompressive craniectomy including C1 laminectomy. Postoperatively, the patient’s neurological exam gradually improved as she awoke and became progressively more alert. One month after presentation, the patient was discharged to rehabilitation moving both upper extremities without difficulty but had decreased movement of the bilateral lower extremities. Follow-up imaging at 2 months demonstrated resolution of cerebellar edema, with resultant bilateral cerebellar encephalomalacia (Fig. 2g). At 10 months after initial presentation, the patient was able to ambulate independently and had neurologically recovered.

## Discussion

The Centers for Disease Control (CDC) reports that in 2021 alone, more than 80,000 people died of an opioid overdose [3]. It is reported that thousands of infants are born each year with opioid withdrawal. Furthermore, toddlers are ingesting caregiver’s medications and children and adolescents are prescribed opioids leading to addictions [4]. The most common place for a pediatric opioid death is in the household and most of these deaths are unintentional. The most common drugs implicated in pediatric overdose deaths were prescription opioids [4]. While the increasing toll of opioid addiction is often discussed among adults, the pediatric population has become a significant victim of the epidemic.

A spectrum of opioid toxicity–related syndromes has been described in the literature. These include CHANTER syndrome, pediatric opioid use-associated neurotoxicity with cerebellar edema (POUNCE) syndrome, heroin-associated spongiform leukoencephalopathy (also known as “chasing the dragon leukoencephalopathy”), and opioid-associated amnestic syndrome (OAA). While all these toxidromes share many similarities, they can be distinguished from one another by differences on imaging.

POUNCE syndrome has previously been described in children accidentally exposed to opioids who presented with altered mental status [5]. On imaging, malignant cerebellar edema is a prominent finding which often results in obstructive hydrocephalus similar to CHANTER syndrome. POUNCE syndrome is not associated with involvement of the hippocampi and basal ganglia, unlike CHANTER syndrome (as seen in our case). POUNCE syndrome is characterized by its predilection for white matter with areas of hypoattenuation on CT, and T2 hyperintense lesions on MRI [1]. Similar cases of accidental pediatric overdoses with subsequent cerebral edema have also shown a pattern of leukoencephalopathy rather than hippocampal and basal ganglia injury [6, 7]. This is hypothesized to be due to differing distribution and predominance of opiate receptors between adults and children [8].

Clinically, pediatric opioid intoxication manifests similarly to adults. The most common presenting symptoms include drowsiness, vomiting, respiratory depression, miosis, agitation, and tachycardia [9]. Opioid overdose in children differs from the adult population in that there can be a delayed onset of toxicity, unexpectedly severe poisoning, and prolonged toxic effects. These can be attributed to the fact that children have differing rates of drug absorption and differing metabolisms, and drugs may distribute more easily into the central nervous system of children [10]. Furthermore, children often ingest higher doses than adults per kilogram of body weight and are less likely to have any opioid tolerance [11].

Diffuse cerebellar edema with restricted diffusion and crowding of the fourth ventricle are hallmark imaging findings of CHANTER syndrome [12]. This pattern of restricted diffusion was found in our 2-year-old patient after a fentanyl overdose, who presented with cerebellar edema and fourth ventricular compression. Vascular imaging ruled out arterial occlusion as the etiology of cerebellar edema and the patient’s history of fentanyl consumption made CHANTER syndrome the likely diagnosis. Fortunately, the patient did not further suffer from venous infarction, although there was imaging evidence of venous hypertension. Yet, we postulate that the deep ganglionic and hippocampal signal changes encountered in these patients are perhaps related to venous hypertension, which was potentially present in our case.

## Conclusions

This case report presents a young child who was diagnosed with CHANTER syndrome and ultimately survived. With the use of opioids evermore increasing, identifying, diagnosing, and treating the various opioid toxidromes, especially in children, are of paramount importance. We hope that our case increases the awareness of this entity.
